# Cryptochromes in mammals: a magnetoreception misconception?

**DOI:** 10.3389/fphys.2023.1250798

**Published:** 2023-08-21

**Authors:** Li Zhang, E. Pascal Malkemper

**Affiliations:** Max Planck Research Group Neurobiology of Magnetoreception, Max Planck Institute for Neurobiology of Behavior—caesar, Bonn, Germany

**Keywords:** magnetic sense, chronobiology, magnetic fields, radical pair, spatial orientation

## Abstract

Cryptochromes are flavoproteins related to photolyases that are widespread throughout the plant and animal kingdom. They govern blue light-dependent growth in plants, control circadian rhythms in a light-dependent manner in invertebrates, and play a central part in the circadian clock in vertebrates. In addition, cryptochromes might function as receptors that allow animals to sense the Earth’s magnetic field. As cryptochromes are also present in mammals including humans, the possibility of a magnetosensitive protein is exciting. Here we attempt to provide a concise overview of cryptochromes in mammals. We briefly review their canonical role in the circadian rhythm from the molecular level to physiology, behaviour and diseases. We then discuss their disputed light sensitivity and proposed role in the magnetic sense in mammals, providing three mechanistic hypotheses. Specifically, mammalian cryptochromes could form light-induced radical pairs in particular cellular milieus, act as magnetoreceptors in darkness, or as secondary players in a magnetoreception signalling cascade. Future research can test these hypotheses to investigate if the role of mammalian cryptochromes extends beyond the circadian clock.

## Introduction

Many animals perceive the weak magnetic field of our planet, a modality called magnetoreception (Nordmann et al., 2017). A magnetosensitive organ still awaits discovery, and there have been multiple different hypotheses, including mechanisms based on a physical alignment sensor (magnetite), electromagnetic induction, and a quantum sensor. Thus far, a strong line of evidence supports a quantum sensory mechanism based on light-induced radical pairs in birds (Hore and Mouritsen, 2016). Currently, the most likely candidates for the receptors are proteins from the cryptochrome family (Hochstöger et al., 2020), which is also present in mammals. Are mammalian cryptochromes also possible candidates for a magnetosensor?

Cryptochromes are structural homologs of photolyases (Sancar, 2004), enzymes that repair UV-B light-induced DNA damage using the energy from blue and UV-A light (Sancar, 2003). However, whilst still containing the photolyase homology regions (PHR), cryptochromes no longer have DNA repair functions. Instead, they have developed new signalling roles, mediated through highly dynamic C-terminal extensions (Sancar, 2003; Brautigam et al., 2004; Zhu et al., 2005; Chaves et al., 2006; Huang et al., 2006; Chaves et al., 2011; Zoltowski et al., 2019; Parico et al., 2020).

Modern classification distinguishes plant CRYs, which are blue light photoreceptors (Ahmad and Cashmore, 1993; Wang et al., 2016; Wang and Lin, 2020) and animal CRYs. Animal CRYs are further subdivided into *Drosophila* type CRY (dCRY or Type I CRY), Type II CRYs, and Type IV CRYs (Chaves et al., 2011). Type IV CRYs and dCRY are photoreceptors that mediate light responses such as circadian clock entrainment and putatively light-dependent magnetoreception (Stanewsky et al., 1998; Gegear et al., 2008; Zhang et al., 2017; Hochstöger et al., 2020; Xu et al., 2021). In contrast, Type II CRYs do not appear to fulfil photoreceptive functions but act as light-independent clock genes in the circadian transcription-translation feedback loop (TTFL) that orchestrates cellular and behavioural rhythms (Griffin et al., 1999; Kume et al., 1999; Shearman et al., 2000). While a magnetic sense in mammals is established (Burda et al., 2020), the involvement of mammalian cryptochromes has been questioned because they only express Type II CRYs (Kavet and Brain, 2021). In the following, we look at the evidence for and against a connection between Type II CRYs and the magnetic sense.

## Structure and function of mammalian cryptochromes

Mammals possess two CRYs, CRY1, and CRY2, which have been studied mainly in humans and mice (Hsu et al., 1996; Todo et al., 1996; Kobayashi et al., 1998). The two human CRYs share 73% amino acid sequence identity, with the highest sequence divergence in the C-terminal tail (CTT) region (Hsu et al., 1996). CRYs are found across all tissues, but relative expression levels vary between the two proteins (van der Spek et al., 1996; Kobayashi et al., 1998). CRY1 is expressed at high levels in the central circadian pacemaker, the suprachiasmatic nucleus (SCN), where it oscillates in a circadian manner (Miyamoto and Sancar, 1998; Kume et al., 1999). CRY2 levels, on the other hand, are higher in the retina (Miyamoto and Sancar, 1998; Thompson et al., 2003). The mammalian retina is an independent circadian oscillator that entrains to light-dark cycles (Tosini and Menaker, 1996). The photoentrainment is mediated by Neuropsin (Buhr et al., 2015), but CRYs have been suggested to be involved (Vanderstraeten et al., 2020). CRY mRNA was found across all layers of the retina (Miyamoto and Sancar, 1998; Thompson et al., 2003), but immunohistochemistry detected retinal CRY2 exclusively in the photoreceptor layer and CRY1 in cones, amacrine cells and some ganglion cells (Wong et al., 2018).

**FIGURE 1:**
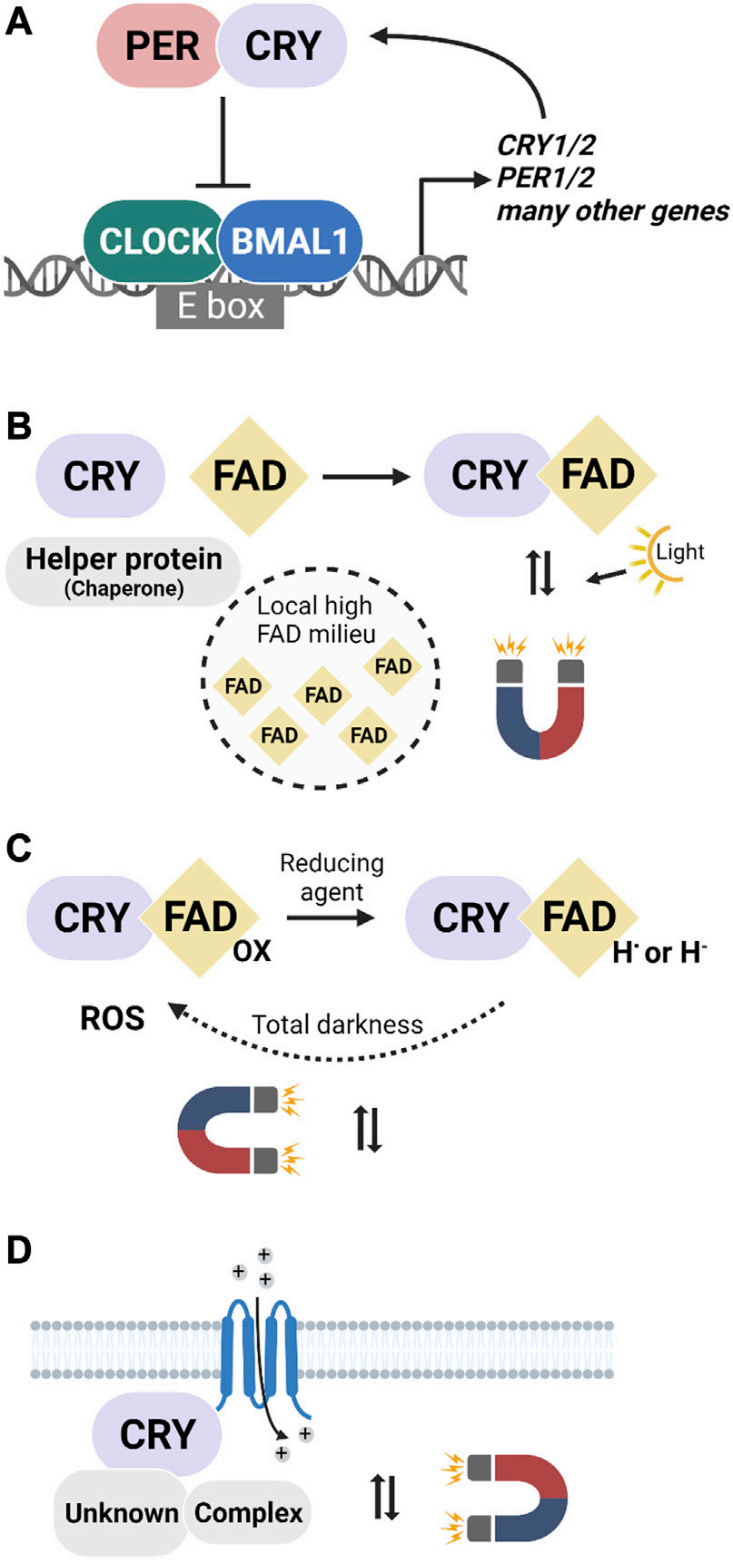
The canonical function of mammalian cryptochromes and three hypothetical scenarios for CRY involvement in magnetic sensing. **(A)** The well-known role of CRYs in the transcription-translation feedback loop (TTFL) where they control rhythmic gene expression in a light-independent manner. **(B–D)** Three putative ways in which mammalian cryptochromes could be involved in the transduction of magnetic field effects. **(B)** Light-dependent magnetosensitivity in a specific cellular milieu. FAD-binding could be facilitated by, e.g., helper proteins or high FAD concentrations. **(C)** Light-independent activation by a reducing agent and re-oxidation in the dark accompanied by radical pair production. **(D)** CRYs as secondary (non light-sensitive) components of a magnetosensory signaling cascade that would be initiated by a primary radical pair-forming (photoreceptor) protein that is currently unknown.

Mammalian CRYs are central players in the TTFL that controls cellular circadian rhythms across tissues (Figure 1A). At the core of the loop are the transcription factors CLOCK-BMAL1 that activate many genes, including those of CRYs and PERIOD (PER) proteins (Partch et al., 2006; Dardente et al., 2007). In the cytosol, CRYs form heterodimers with PER and translocate back into the nucleus (Smyllie et al., 2022), where they inhibit CLOCK-BMAL1 and their own expression, before CRY and PER degradation starts another 24 h cycle (Kume et al., 1999). CRY1 and CRY2 appear to play similar and partially redundant roles in the TTFL, but different repression potency for the CLOCK-BMAL1 complex results in distinct antagonistic circadian phenotypes in single CRY1 or CRY2 knockout mice (van der Horst et al., 1999; Vitaterna et al., 1999; Anand et al., 2013; Miller and Hirota, 2022). In total darkness, CRY1 and CRY2 knockout mice have 1-h faster or slower free-running rhythms of locomotor activity, respectively, while double-knockouts are arrhythmic (Thresher et al., 1998; van der Horst et al., 1999).

The central role of CRYs in the TTFL in nearly every cell of the body has far-reaching consequences. For example, apart from their role in the central pacemaker, CRYs are involved in the circadian rhythmicity of the autonomous nervous system and insulin metabolism (Ikeda et al., 2007) and the regulation of autoimmunity (Cao et al., 2017). Consequently, perturbations of the CRY genes can manifest in various diseases. Genetic studies in humans have linked CRY mutations with mood disorders (Soria et al., 2010; Kovanen et al., 2013; Geoffroy et al., 2015), sleep disorders (Kripke et al., 2015; Hirano et al., 2016; Parico et al., 2020), and metabolic syndrome (Kovanen et al., 2015). The disruption of circadian rhythms increases the risk of some forms of cancer (West and Bechtold, 2015) and CRYs appear to be a direct link (Chan and Lamia, 2020). Various pro- and anti-cancerogenic effects of CRYs, including the promotion of genomic stability (Hoffman et al., 2010), induction of cell proliferation in human breast cancer cells (Chun et al., 2015), as well as the reduction of tumour formation and growth (Huber et al., 2016; Dong et al., 2019), have been reported. While still in its infancy, the field of circadian medicine, in particular the search for specific small molecule activators or repressors of mammalian cryptochromes (Miller and Hirota, 2022) and photopharmacological regulation of the circadian clock (Kolarski et al., 2021a; Kolarski et al., 2021b), are emerging fields of large potential (Kramer et al., 2022).

## The role of cryptochromes in the magnetic sense

The organ(s) that allows animals to detect magnetic fields still awaits discovery, but a line of research on birds supports a quantum sensory mechanism based on radical pairs (Hore and Mouritsen, 2016). The influence of weak magnetic fields on radical pairs is well-known and has been associated with a variety of biological effects (Zadeh-Haghighi and Simon, 2022). The radical pair mechanism (RPM) predicts that the magnetic modulation of a spin-dependent chemical reaction initiates a transduction cascade, ultimately leading to the generation of neuronal signals (Hore and Mouritsen, 2016). Cryptochromes have been proposed as the primary receptors for this mechanism because they form intramolecular radical pairs within their photocycle (Ritz et al., 2000). This photocycle is best studied in *Arabidopsis*, where light excites the flavin adenine dinucleotide (FAD) cofactor and induces an electron transfer along a series of conserved tryptophan residues, resulting in the reduction of FAD and the formation of a radical pair between the terminal tryptophan and FAD (Chaves et al., 2011). The FAD reduction leads to a conformational change of the protein, resulting in a signalling state that initiates downstream effects (Banerjee et al., 2007). In the presence of oxygen, FAD will then be re-oxidised to the ground state, a process that occurs in darkness and involves the formation of a second pair of radicals (Pooam et al., 2019). In non-mammalian vertebrates, CRY4 is a magnetoreceptor candidate, although the exact cellular localization is still debated (Günther et al., 2018; Pinzon-Rodriguez et al., 2018; Hochstöger et al., 2020). It binds stoichiometric amounts of FAD *in vitro*, forms light-dependent radical pairs, and changes its conformation after light excitation (Watari et al., 2012; Mitsui et al., 2015; Zoltowski et al., 2019; Hochstöger et al., 2020; Xu et al., 2021). This provides a compelling case for CRY4, although some experimental findings such as magnetic orientation under monochromatic green light (Wiltschko et al., 2010) or when the magnetic field and light are presented alternatingly (Wiltschko et al., 2016) are not consistent with the canonical RPM model. Independent of the underlying receptor, a diagnostic test for the involvement of radical pairs is investigating magnetic behaviours in the presence of weak radiofrequency (RF) magnetic fields that are expected to interfere with the interconversion of the radicals (Henbest et al., 2004) and migratory birds are disoriented in such fields (Engels et al., 2014; Leberecht et al., 2023).

Sensitivity to magnetic fields has also been reported in mammals such as bats, mole-rats, mice, and humans (Burda et al., 2020). Some mammals, such as subterranean mole-rats, appear resistant to RF, so their magnetic sense is unlikely to involve radical pairs (Thalau et al., 2006). Interestingly, though, in epigeic mammals such as hamsters, mice, and humans, RF effects on magnetic responses have recently been reported (Malkemper et al., 2015; Malewski et al., 2018; Painter et al., 2018; Chae et al., 2022; Phillips et al., 2022; Phillips and Painter, 2023). If these findings are correct, the question of receptor identity arises. It is possible that mammalian magnetoreceptors evolved independently of birds and invertebrates, and alternative receptors such as melanopsin have been suggested (Phillips et al., 2022), but some evidence points to an involvement of CRYs.

Support for magnetic sensitivity of mammalian CRYs is provided by studies of heterologous expression of recombinant Type II CRY in insects. For example*,* UV/blue light-dependent magnetic behaviours were reported in the fruit fly *Drosophila melanogaster* (Gegear et al., 2008; Yoshii et al., 2009; Bassetto et al., 2023). The response was abolished by knocking out dCRY and rescued by the expression of insect Type II CRY or human CRY2 (but not CRY1; Gegear et al., 2010; Foley et al., 2011; Fedele et al., 2014a). Light-dependent magnetic responses consistent with an RPM were also found in cockroaches and firebugs, although both insects only possess vertebrate Type II cryptochromes (Bazalova et al., 2016; Netusil et al., 2021).

In sum, there is evidence for a magnetic sense in mammals and for a relationship between magnetic field responses and cryptochromes in other animals. At the core of the canonical RPM lies the formation of a light-induced radical pair between the CRY protein and FAD, leading to the question if mammalian CRYs are photosensitive.

## Are mammalian cryptochromes photoreceptors?

Due to their similarity to photolyases and the light-sensitive CRYs from *Arabidopsis* and *Drosophila*, it was initially assumed that mammalian CRYs are also photoreceptors. Their role in the circadian clock seemed to support this assertion: To keep in sync with the outside world, the internal clock resets to light stimuli which, in *Drosophila,* is mediated by dCRY (Emery et al., 1998; Stanewsky et al., 1998; Ishikawa et al., 1999). Light input through the retina also resets the mammalian circadian clock (Shigeyoshi et al., 1997) and CRYs are expressed in the retina (Thompson et al., 2003; Wong et al., 2018). Unexpectedly, however, while CRY1/2 double-knockout mice are arrhythmic in total darkness, light pulses still induced phase shifts in the SCN (Okamura et al., 1999) and behavioural entrainment to a light/dark cycle (van der Horst et al., 1999). Thus, CRYs do not reset the circadian clock. A series of elegant experiments demonstrated that melanopsin, in intrinsically photoreceptive ganglion cells, fulfils that function and CRYs are neither sufficient nor necessary (Berson et al., 2002; Hattar et al., 2002; Hattar et al., 2003). Other non-visual photoreceptive responses in mammals that have been discussed to be related to CRY photoreception—circadian rhythms of electroretinograms, contrast sensitivity and pupillary light responses (Van Gelder et al., 2003; Vanderstraeten et al., 2020)–are also consistent with circadian gating by CRYs, i.e., the modulation of input from other photoreceptors (Wong et al., 2018). Finally, mutation analyses demonstrated that the conserved tryptophan residues essential for photoreception in other species are dispensable for the circadian function in mammals (Froy et al., 2002). In sum, mammalian CRYs do not appear to function as photoreceptors in the circadian clock.

It is possible that vertebrate Type II CRYs have lost their capability to sense light during evolution. The photosensitivity of CRYs depends on FAD that is sequestered non-covalently in a U-shaped binding pocket in the PHR (Zoltowski and Gardner, 2011). The motifs of the pocket that are critical for FAD binding are known from crystal structures of plant CRY (Brautigam et al., 2004), dCRY (Czarna et al., 2013), and pigeon CRY4 (Zoltowski et al., 2019). No full-length crystal structures of mammalian cryptochromes are available to date. Partial structures of mouse CRYs show that in contrast to dCRY where a phosphate-binding loop forms a lid that traps FAD in the binding pocket (Maul et al., 2008), the binding pocket of mouse CRY has a more open conformation that exposes the cofactor and enables dynamic FAD-binding (Nangle et al., 2013; Xing et al., 2013). Accordingly, computer simulations predicted a strikingly reduced binding affinity for FAD in mouse CRYs (Kutta et al., 2017). The authors identified the structural features responsible for the reduced affinity and, based on sequence alignments, concluded that all animal Type II CRYs are only vestigial flavoproteins. They estimated that *in vivo* less than 7% of human CRY2 and 16% of CRY1 have FAD bound (Kutta et al., 2017).

Accordingly, only low (sub-stoichiometric) levels of FAD have been reported for purified animal Type II CRYs (Ozgur and Sancar, 2003; 2006; Kutta et al., 2017; Wang et al., 2018; Hochstöger et al., 2020; Bolte et al., 2021). In contrast, other cryptochromes, such as CRY4, can readily be purified with FAD bound (Hochstöger et al., 2020; Xu et al., 2021).

In sum, all investigated mammals express animal Type II CRYs with very low binding affinity for FAD *in vitro*. Thus, in the absence of evidence for Type I or Type IV CRYs in mammals, it appears unlikely that mammalian cryptochromes are photosensitive. Can the low binding affinity to FAD be reconciled with the findings of Type II CRY as part of a magnetoreception system?

## Scenarios for mammalian cryptochrome magnetoreception

We can think of three, not mutually exclusive, scenarios in which mammalian cryptochromes could be involved in magnetoreception: 1) The cellular milieu facilitates FAD binding, 2) Cryptochromes act as magnetoreceptors in darkness, or 3) Cryptochromes are downstream interactors in a magnetoreception signalling cascade.

First, although evidence argues against a general photosensitivity of animal Type II CRYs, it conceivably may occur in a specific cell type or subcellular compartment (Figure 1B). Direct support for FAD-binding to mammalian CRYs is provided by a study on FBXL3, a ubiquitin ligase that binds to the FAD pocket and labels cryptochrome for proteasomal degradation. FAD directly competed with FBXL3 for binding and thereby reduced CRY degradation (Hirano et al., 2017). Further indirect structural evidence is that a fourth tryptophan residue, which is suggested to be fundamental to generating the light-induced signalling state in bird CRY4-mediated magnetoreception (Xu et al., 2021), is also conserved in humans and mice (Muller et al., 2015). Finally, human CRY1 (expressed in flies) undergoes light-dependent proteolysis, indicative of conformational changes and the formation of radicals (Hoang et al., 2008).

Dynamic, rather than static, association with FAD is being recognized as a common phenomenon in flavoproteins (Schnerwitzki and Vabulas, 2022). It follows that, although the affinity for FAD is much weaker in Type II than in Type I/IV CRYs, specific cellular conditions (e.g., redox conditions, high compartmental FAD concentrations or the presence of binding partners) could augment FAD binding to Type II CRYs. In support of this, successful purifications of Type II CRYs with FAD were reported (Hsu et al., 1996; Liedvogel et al., 2007). Interestingly, the very few studies that reported stoichiometric FAD binding supplemented the culture media with FAD, suggesting that high FAD concentrations compensate for the low binding affinity of Type II CRYs (Table 1). Mammals cannot synthesize riboflavin, the precursor of FAD, they must take it up from external sources (Powers, 2003). Intracellularly, a complex of enzymes converts riboflavin into FAD. Consequently, FAD concentrations are not homogenous within a cell, but they vary across time, between cell types or states and across subcellular compartments (Giancaspero et al., 2013; Hirano et al., 2017). A particularly striking example is the 15-fold increase of intracellular FAD during adipogenic differentiation of mouse fibroblasts (Hino et al., 2012). *In vivo*, conditions favouring FAD binding could prevail in specific cell types or compartments, such as the outer segments of photoreceptors in which a Type II CRY (CRY1a) is enriched in some birds and mammals (Niessner et al., 2011; Niessner et al., 2016; Bolte et al., 2021). Within these cells, FAD concentrations would have to be in the higher micro-molar range (CRY1: >16 µM, CRY2: >40 µM) to reach sufficient saturation of Type II CRYs (Kutta et al., 2017). Free FAD concentrations in mammalian cells, however, are only in the nanomolar range, but eight to ten-fold differences were found between cell lines and organs (Hühner et al., 2015). We could not find systematic data on FAD concentrations across different cell types and interspecies differences *in vivo*.

**TABLE 1:** Published attempts to purify vertebrate Type II cryptochromes with FAD in heterologous expression systems (in chronological order). (YES) indicates not reported or sub-stoichiometric FAD binding levels.

Host species	CRY1/2	Expression system	Promoter	FAD bound?	FAD supplement?	Stoichiometric?	Method of analysis	References
*Homo sapiens*	hCRY1, hCRY2	Bacterial	Ptac	(YES)	NO	Not reported	UV-vis absorption spectroscopy	Hsu et al. (1996)
*Homo sapiens*	hCRY2	Mammalian cell line (Hela)	CMV	(YES)	NO	NO (∼30%)	Fluorescence spectroscopy	Özgür and Sancar (2003)
*Homo sapiens*	hCRY1, hCRY2	Virus/insect cell (Sf21)	CMV	(YES)	NO	NO (<0.1% for hCRY1, ∼0.2% for CRY2)	Fluorescence spectroscopy	Özgür and Sancar. (2006)
*Sylvia borin*	sbCRY1a	Virus/insect cell (Sf9)	CMV	YES	YES (25 µM)	YES	UV-vis absorption spectroscopy	Liedvogel et al. (2007)
*Homo sapiens*	hCRY1	Virus/insect cell (Sf21)	AcMNPV	(YES)	NO	Not reported	Fluorescence spectroscopy	Hoang et al. (2008)
*Mus musculus*	mCRY1	Mammalian cell line (HEK293)	CMV	(YES)	YES (500–5,000 µM)	Not reported	Pull-down assay	Hirota et al. (2012)
*Homo sapiens*	hCRY1	Yeast (Pichia pastoris)	UAS	YES	YES (40 µM)	YES	EPR spectroscopy	Vieira et al. (2012)
*Mus musculus*	mCRY2	Virus/Insect cell (High5)	CMV	YES	YES (500 µM)	YES	Fluorescence spectroscopy	Xing et al. (2013)
*Sylvia borin*, *Homo sapiens*	sbCRY1a, hCRY1, hCRY2	Bacterial	T7	NO	NO	NO	UV-vis absorption spectroscopy	Kutta et al. (2017)
*Columba livia*	clCRY1	Virus/insect cell (Sf21)	AcMNPV	NO	NO	NO	UV-vis absorption spectroscopy	Wang et al. (2018)
*Columba livia*	clCRY1a, clCRY2a	Virus/insect cell (Sf9)	Polyhedrin	NO	NO	NO	UV-vis absorption spectroscopy	Hochstöger et al. (2020)

Apart from FAD concentrations, protein interactions could further facilitate FAD binding. The structure of the highly conserved phosphate-binding loop of the FAD binding pocket is sensitive to the local environment, which led to the suggestion that protein-protein interactions might increase the affinity of FAD (Nangle et al., 2013). Specifically, one enzyme of the FAD-producing pathway, FAD synthetase, was suggested to act as a chaperone that delivers the flavin cofactor to apoproteins (Torchetti et al., 2011; Giancaspero et al., 2015).

Summarizing this first scenario, cellular and subcellular differences in FAD concentrations and the availability of binding partners could have a significant effect on the fraction of CRYs with FAD bound. Under certain conditions, mammalian CRYs might form light-induced radical pairs.

In a second scenario, mammalian CRYs would be part of a radical pair mechanism independent of light (Figure 1C). Indeed, Type II CRY-dependent magnetic behavioural responses occur after 24 h in total darkness (Netusil et al., 2021). These findings are inconsistent with the canonical RPM and suggest that the radicals formed during the reoxidation of the anionic flavin radical state provide the magnetosensory step of the CRY cycle (Wiltschko et al., 2016; Pooam et al., 2019). In dCRY, this step occurs in darkness and within minutes, but in mammals, the anionic radical appears more stable. Heterologously expressed human CRY1 exists in a mix of redox states with a significant portion of the anionic radical state in the absence of light (Vieira et al., 2012). In *Drosophila*, chemical reduction can replace photoreduction of FAD for dCRY activation (Vaidya et al., 2013). It follows that in a reducing intracellular environment, a significant proportion of CRY would be in the anionic flavin radical state, which, in the presence of oxygen, can revert to the ground state and form radical pairs in darkness (Hoang et al., 2008). A side effect of this reaction is the production of reactive oxygen species (Arthaut et al., 2017). Consistent with this, pulsed magnetic fields were reported to increase reactive oxygen species levels in mammalian cells in a cryptochrome-dependent manner (Sherrard et al., 2018). ROS might even constitute part of a signalling mechanism (El-Esawi et al., 2017). In sum, it is conceivable that chemical, rather than photochemical reduction, could lead to the generation of magnetosensitive radical pairs in mammalian cryptochromes.

Finally, CRYs could play a role in the magnetic sense even without forming radical pairs themselves (Figure 1D). Instead of being the primary receptor molecules, they may be a downstream component of a signalling cascade. After all, mammalian CRYs have highly dynamic C-terminal domains, indicative of evolutionarily optimized protein-protein interactions. Consistent with a secondary role for cryptochromes, studies in *Drosophila* showed that the FAD-binding domain and a series of tryptophans, which are required to form the radicals in the canonical RPM hypothesis, are not necessary for CRY to convey magnetosensitivity (Fedele et al., 2014a; Fedele et al., 2014b; Bradlaugh et al., 2023). The authors speculated that the magnetosensitive radical pairs do not form within CRYs, instead suggesting a role for redox coupling between FAD and a redox-sensitive potassium channel (Fogle et al., 2015). Indeed FAD-dependent magnetic field effects, albeit in mT-strong fields, were reported for mammalian cells (Ikeya and Woodward, 2021). In this scenario, CRYs would be part of a magnetoreceptor complex without forming radical pairs themselves.

**Box 1 Future Issues**

• Is the finding of an RPM-based magnetic sense in mammals robust?
• Do some mammals express Type I or IV cryptochromes?
• Are there binding partners of mammalian cryptochromes that are not involved in circadian rhythms?
• Do small molecule cryptochrome inhibitors perturb magnetoreception?
• If cryptochromes are not directly responsible for magnetic field sensitivity, what are alternative candidate molecules?

## Conclusion

Mammalian cryptochromes are light-independent players in the mammalian circadian clock, but they might also serve functions beyond this. Based on evidence for magnetic field effects in mammals we put forward three hypotheses describing how CRYs might be related to these effects. Although these hypotheses may prove to be incorrect, we believe it is important to test them. Given the many physiological processes in which cryptochromes are involved in mammals, magnetic sensitivity would have profound implications. The right questions (authors’ choice in Box 1) and experiments will clarify whether CRY-mediated magnetoreception in mammals is a possibility or misconception.
